# Pain-Relieving Effects of Shockwave Therapy for Ledderhose Disease: An Ultrasound-Based Study of an Unusual Bilateral Case

**DOI:** 10.3390/life14020169

**Published:** 2024-01-24

**Authors:** Federica Fulceri, Larisa Ryskalin, Gabriele Morucci, Francesco Busoni, Paola Soldani, Marco Gesi

**Affiliations:** 1 Department of Translational Research and New Technologies in Medicine and Surgery, University of Pisa, 56126 Pisa, Italy; federica.fulceri@unipi.it (F.F.); larisa.ryskalin@unipi.it (L.R.);; 2 Center for Rehabilitative Medicine “Sport and Anatomy”, University of Pisa, 56121 Pisa, Italy

**Keywords:** extracorporeal shock wave therapy, plantar fibromatosis, Ledderhose disease, benign disease, hyperproliferation, plantar fascia, ultrasound-based imaging

## Abstract

Ledderhose disease (LD, or plantar fibromatosis) is a rare, nodular, hyperproliferative condition affecting the plantar aponeurosis of the foot. At present, several conservative, non-surgical treatments have been documented, although with various degrees of success, with little evidence in the literature supporting their efficacy. In this scenario, extracorporeal shock wave therapy (ESWT) has emerged as a safe, effective, and less invasive approach for the successful treatment of several refractory musculoskeletal conditions and soft tissue injuries. Again, recent experimental evidence has shown that ESWT can exert beneficial effects on different fibroproliferative diseases, including Dupuytren’s and Peyronie’s disease. In contrast, the literature regarding the use of ESWT for LD is extremely limited, and no optimal application parameters have been defined to ensure its effectiveness for this disease. Therefore, in the present paper, we report a case of a 48-year-old male patient who developed bilateral foot LD, which was successfully treated with a novel ESWT protocol of treatment consisting of three sessions at 1-week intervals, with 2000 impulses at 5 Hz with an energy flux density of 0.20 mJ/mm^2^. Our data show that this ESWT treatment protocol was effective in completely relieving pain, restoring full functional activity, and thus, greatly improving the patient’s quality of life.

## 1. Introduction

**Figure 1:**
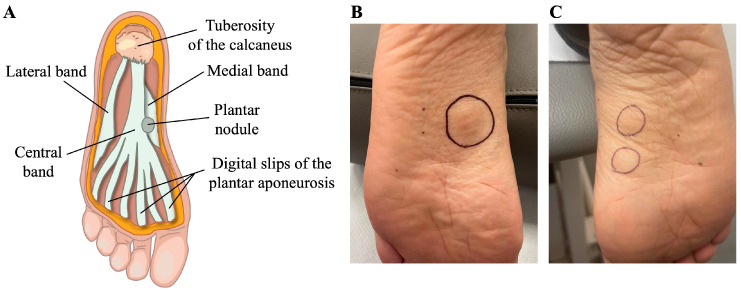
Clinical presentation of a bilateral case of plantar fibromatosis. (**A**) Schematic representation of the gross anatomy of the plantar aponeurosis showing a subcutaneous nodule. Gross appearance of the patient’s feet showing (**B**) a firm, painful protruding nodule on the medial plantar aspect of the left foot and (**C**) two small asymptomatic nodules on the medial plantar aspect of the right foot. Nodules are highlighted by black circles.

Ledderhose disease (LD), or plantar fibromatosis, is a rare benign, hyperproliferative condition affecting the plantar aponeurosis (PA) of the foot [1]. First described in 1894 by Dr. George Ledderhose [2], this condition is characterized by the formation of one or more subcutaneous slow-growing and painless nodules within both the medial and central band of PA [3] along the medial longitudinal arch of the sole (Figure 1A). Even if, initially, patients are asymptomatic, complaints of pain may occur when standing or walking for longer periods. Furthermore, as the nodules enlarge, they become markedly tender, erythematous, and painful and evolve into debilitating lesions that negatively affect the patient’s ability to bear weight and their overall quality of life. The occurrence of multiple nodules frequently occurs over time, thus exacerbating the patient’s discomfort. Bilateral involvement occurs only in 25% of LD cases [4,5].

Although its precise etiology and prevalence are not yet understood, this disorder frequently affects middle-aged and elderly adults, and it is more common in men than women [6]. Furthermore, LD often occurs concomitantly with other fibroproliferative diseases, including Dupuytren’s (DD) and Peyronie’s disease (PD), affecting the palmar aponeurosis of the hand and the tunica albuginea of the penis, respectively [3].

At present, there are several conservative treatments for LD with varying degrees of scientific evidence of their effectiveness. These include anti-inflammatory drugs, orthotics and custom-made soles, physical therapy, and radiotherapy [6,7,8]. Surgical management is advocated for severe cases refractory to conservative treatments. Three main techniques are generally adopted, which include local excision, wide excision, or complete fasciectomy. However, several reports indicate that each of these surgical interventions has a high nodular recurrence rate and multiple wound complications [6,7,8].

Over the last few years, extracorporeal shock wave therapy (ESWT) has been brought increasingly into focus as a safe, effective, and advantageous alternative for the treatment of different musculoskeletal and orthopedics disorders, including plantar fasciitis, Achilles’ tendinopathy, lateral epicondylitis, non-calcific supraspinatus tendinopathy and calcifying tendonitis of the shoulder alongside the delayed union and non-union of long bone fractures [9,10,11,12]. Again, satisfactory results were obtained from ESWT’s application for the treatment of different progressive fibroproliferative conditions, such as DD and PD [13,14,15,16]. On the contrary, despite the similar pattern of disease, the literature regarding the use of ESWT for LD is extremely limited, with different degrees of success [13,17,18]. At the same time, as far as we know, very little has been written on the ultrasonographic appearance of plantar fibromatosis.

Therefore, in the present article, we discuss a case of a male patient with an unusual bilateral LD treated with a modified treatment protocol of focused ESWT to achieve pain-free status and completely restore their functional ability. Furthermore, we report and discuss in detail the ultrasound imaging of both treated (symptomatic) and untreated (asymptomatic) plantar nodules.

## 2. Case Presentation

A 48-year-old man with no significant medical history came to our department complaining of walking difficulties because of a painful protruding nodule on the medial plantar aspect of the left foot (Figure 1B). Unexpectedly, at clinical examination, we detected two relatively small palpable subcutaneous nodules on the right foot (Figure 1C). Unlike the one on the left foot, these latter nodules were totally asymptomatic, and they did not prevent the patient from weight-bearing or walking, nor did they cause pain and discomfort with footwear. A physical examination of both feet revealed no toe contractures or deformities. Furthermore, the patient reported no predisposing factors associated with plantar fibromatosis and no family history of the disease. Again, no DD, PD, epilepsy, or diabetic disease were found, making the unusual condition of our case even more interesting.

An ultrasound (US) evaluation was performed using a portable high-resolution ultrasound machine (Mindray DC-70 EXP, Mindary, Shenzhen, China) to confirm the diagnosis and measure the nodules’ size. The US appearance of the nodule was also assessed. In detail, the US examination of the nodule was performed each time with the patient in a prone position. An experienced radiologist (FB) with more than 30 years of experience in US examination selected the static US images used for the evaluation of both nodules’ size and echogenicity. The size of the nodule was calculated in two dimensions for the dorsoventral (minimum) and anteroposterior (maximum) diameter of the nodule on transverse US images. Measurements of the nodules’ maximum and minimum diameter were carried out with ImageJ software 1.52v.

To assess the echogenicity, the mean grey value within the nodule was measured by drawing a thin line on the outer border (i.e., circumference) of the nodule. In order to avoid any bias due to differences in the exposure of the US images, the mean grey value of the selected nodule was divided by the mean grey value of a circular area, identical in size, within the subcutaneous tissue next to the nodule, which was used as a reference. The nodules were defined as either hypo-echogenic or iso-echogenic with respect to the reference area if the ratio was <1 or 1, respectively.

**Figure 2:**
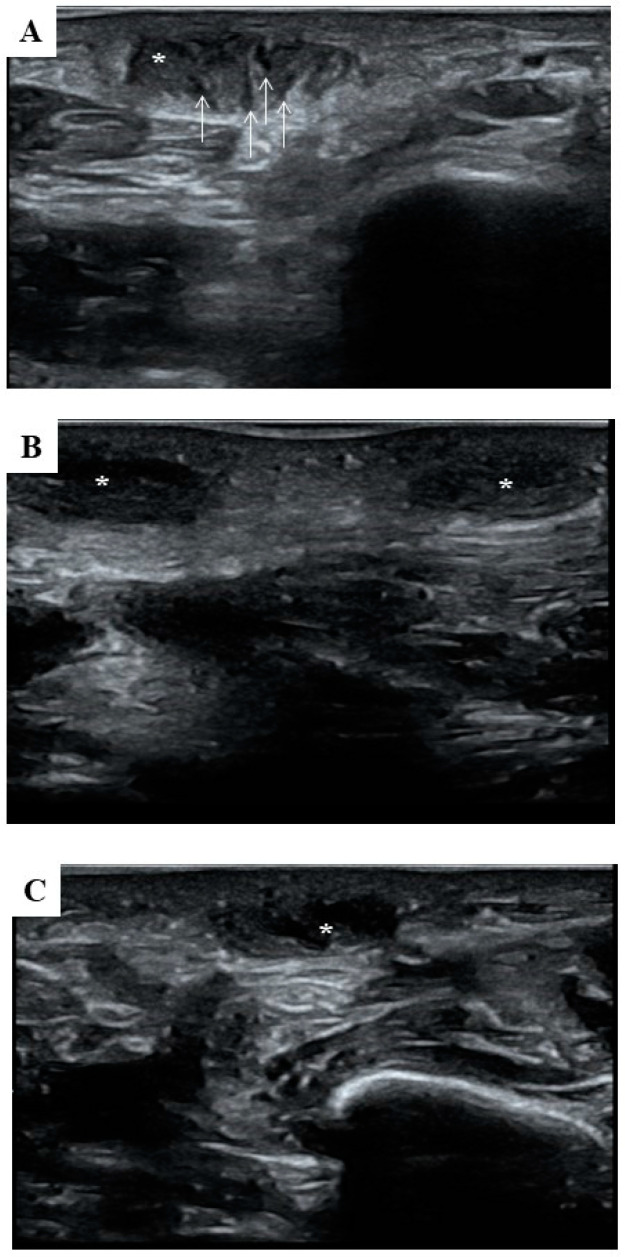
Transverse and longitudinal ultrasonographic images of the plantar nodules. (**A**) Transverse US imaging of the left foot reveals an oval nodule (asterisk) in contiguity to an intact PA. The nodule shows a mixed echogenicity featured by alternating dark and light bands (arrows). (**B**) Longitudinal US imaging of the right foot displaying two close hypoechogenic, small fusiform nodules (asterisks) overlying the PA. (**C**) The transverse US image confirms the hypoechoic and homogeneous structure of the asymptomatic nodule (asterisk) with respect to the surrounding structures and PA.

With reference to the left foot, the US appearance of the symptomatic nodule at the baseline appeared as a well-circumscribed, oval (1.49 ± 0.01 cm and 0.56 ± 0.01 cm), mixed echogenic lesion featuring the “comb sign” [19]. Indeed, within the fibromatosis nodule, an alternation of dark and light linear bands was evident (Figure 2A). On the contrary, an US examination of the plantar aspect of the right foot confirmed the occurrence of two small, fusiform, and homogeneous hypoechoic nodules, measuring 1.07 ± 0.05 cm and 0.42 ± 0.04 cm on average (Figure 2B,C).

Then, the patient underwent ESWT in accordance with the ESWT protocol modified from previous reports [13,17,18]. The Storz DUOLITH^®^ SD1 ultra (Storz Medical AG., Tägerwilen, Switzerland) device was used to administer the treatment once a week for a 3-week period. In detail, 2000 shocks were administered at 5 Hz with an energy flux density of 0.20 mJ/mm^2^. No local anesthesia was applied, and the patient was able to complete treatment with no reported side effects, including local bruising or swelling, transient hematoma, or skin erosion. Of note, the patient expressed satisfaction with the immediate pain-relieving effect after ESWT.

Primary clinical outcomes included the level of subjective pain on a visual analog scale (VAS, 0–10) and self-perceived overall pain and activity limitation with the Roles and Maudsley score (RMS, [20]) (Table 1). The first one is a pain rating scale which consists of a straight line with two endpoints where 0 corresponds to “no pain”, while 10 corresponds to the “most severe intolerable pain”. The other score is a four-point subjective rating scale, which has been used by many investigators when assessing patients’ levels of satisfaction regarding the results of shockwave therapy [21].

Secondary outcomes included the assessment of foot function using an adapted and shortened version of the original Foot Function Index (FFI), namely the 17-Italian Foot Function Index (17-IFFI) [22]. This latter index consists of 17 items divided into three subscales as follows: pain (5 items), disability (9 items), and activity limitation (3 items) to measure the impact of pathologies on foot and ankle function. The global percentage score is calculated with the following equation:

(Sum of subscale scores)/170 × 100 = total score%,

Scores on 17-IFFI range from 0% to 100%, with higher scores indicating a higher degree of disability and worse outcomes [23].

Data were collected at the baseline (T0) and at both immediate (T1) and short-term follow-up (T2), i.e., 1 week and 2 months after the last ESWT session, respectively. 

Statistical analysis was performed with StatView software(version number: 5.0.1). An analysis of variance, ANOVA, followed by Scheffe’s post hoc test, was carried out to compare the differences in the nodules’ size and echogenicity between T0, T1, and T2. Data were expressed as the mean ± standard error. The null hypothesis (H_0_) was rejected for *p* < 0.05.

As reported in Table 2, at the baseline, the patient reported severe pain (6/10 VAS score), functioning as “fair” on the RMS scale (i.e., some discomfort after prolonged activity), and a global score of 34.7% on the 17-IFFI questionnaire.

In detail, with reference to plantar pain, the patient experienced a marked decrease in pain intensity just one week after ESWT treatment, with a reduction in the VAS score from 6/10 at the baseline to 2/10 at T1. Furthermore, at the two-month follow-up visit (T2), he reported the complete resolution of pain (VAS 0/10). Similarly, baseline RMS was markedly improved at immediate and short-term follow-up. In detail, RMS was reduced from 3 points (“fair” grade) at the baseline to 2 points (“good” grade) at T1, and it dropped down to 1 point (“excellent” grade, i.e., no pain, full movement, and activity) at the two-month follow-up (T2). In addition, we observed an important finding in the patient’s positive response regarding the effectiveness of ESWT treatment. Indeed, we observed a remarkable decrease in the 17-IFFI score from 34.7% (T0) to 11.2% at the first follow-up evaluation (T1), reaching the best outcome (0%) two months after the last treatment session (T2). 

**Figure 3:**
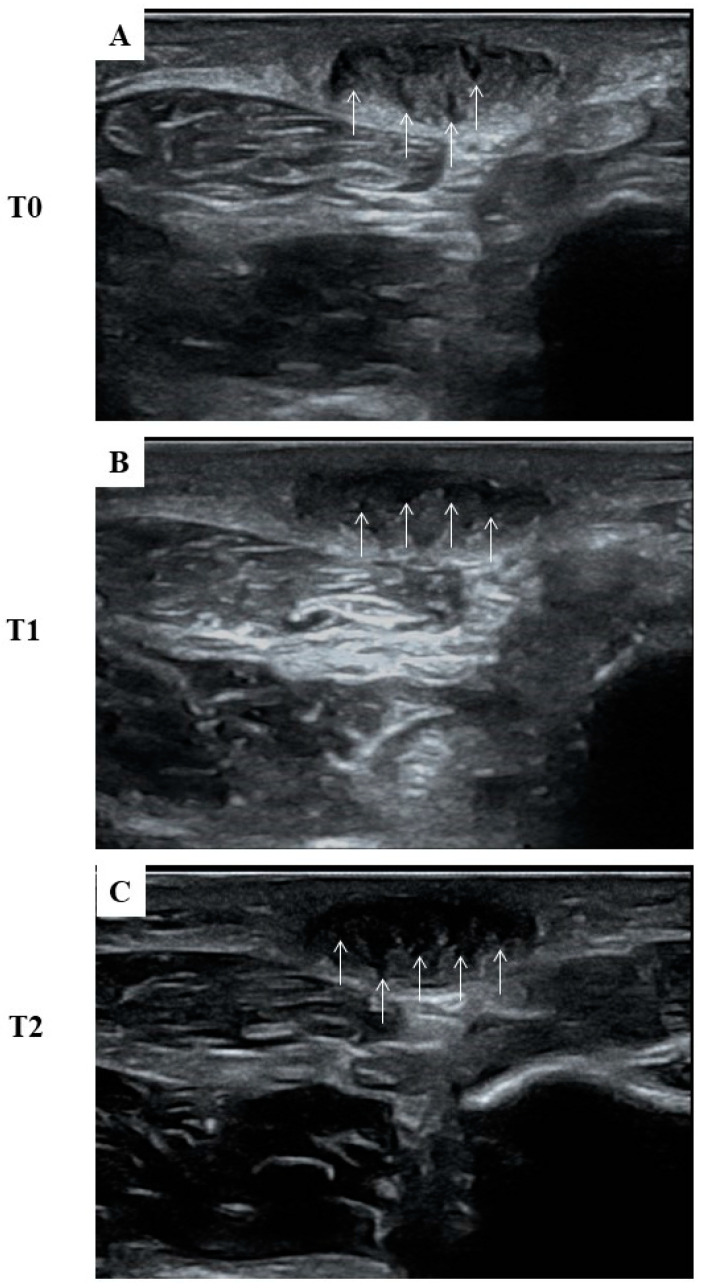
Transverse ultrasonographic images of the left symptomatic nodule at different time intervals. (**A**) Pre-treatment (T0) US examination of the symptomatic nodule revealed a distinctive intralesional feature known as the “comb sign”. This latter, featured by an alternating dark and light linear band, is also evident at (**B**) one week (T1) and (**C**) two months (T2) after ESWT treatment. It can be noted that the dark bands (arrows) within the nodule become progressively larger than the light ones from T0 to T2.

Finally, it is worth mentioning that the decrease in pain level and improvement in functioning were accompanied by a substantial improvement in the softening of the nodule’s consistency, as reported by the patient himself. Despite this, we did not observe any changes in the nodule’s measurement using the US evaluation; after ESWT, the nodules became darker and showed a more uniform pattern on the US from T0 to T2 (Figure 3), which was confirmed by the decrease in the ratio of the mean grey value measured with ImageJ (Table 3).

## 3. Discussion

In our study, we report the immediate and short-term effectiveness of ESWT in reducing pain levels and improving foot function in a patient with LD. 

At present, there is limited evidence in the literature on the beneficial effects of ESWT for LD. Indeed, as far as we know, there are only four previously published articles that have been limited to pilot case series and/or case reports for a total of less than 20 LD feet [13,17,18,24]. In addition, these papers report differing levels of success in terms of pain relief. This may be due to the fact that, at present, no standardized protocol exists for the treatment of plantar fibromatosis. In this regard, the published research protocols show different setup parameters (i.e., dosage, interval of treatments, number of sessions), and different types of equipment are used to generate the shock waves (Table 4). This, in turn, may have substantial influences on achieving ESWT’s therapeutic effects and, thus, good clinical outcomes. 

One of the largest groups of patients treated with ESWT was described by Knobloch and Vogt [13], who reported on the results in six subjects treated with high-energy ESWT applied in two sessions with 7 days between treatments. Despite a significant decrease in pain levels occurring after 14 days, pain (although very mild) still persisted at the three-month follow-up. Lately, Zachariou et al. [18] reported the beneficial effects of high-energy ESWT on a patient with a bilateral presentation of nodules. Even in this case, the ESWT application, with the very same protocol of treatment as Knobloch and Vogt, did not completely relieve symptoms and pain. Indeed, some mild symptoms and pain persisted at the 12-month follow-up, albeit without producing a significant gait impairment. Of note, the patient had intense pain during each shockwave therapy session, which may be due to the very high levels of energy applied to the plantar aspect of the foot. Additionally, it is worth mentioning that, in this latter paper [18], ESWT was administered in combination with capacitive and resistive electric transfer (TECAR) therapy and non-steroidal anti-inflammatory drugs (NSAIDs). As reported in clinical practice, it is generally recommended to avoid the use of NSAIDs for breakthrough pain, as key aspects of the inflammatory cascade may contribute to tissue healing [25,26]. Therefore, patients are advised to stop using painkillers two weeks prior to their first ESWT session, throughout treatment, and at least six weeks after the final ESWT session. Thus, allowing the patient to use NSAIDs prior to and during the treatment may explain why a complete resolution of symptoms after ESWT was not achieved by the authors [18,24]. Comparable results were reported by Frizziero et al. [17], who provided four sessions of treatment at weekly intervals at lower energy flux density, with a maximum peak of 0.20 mJ/mm^2^, according to the patient’s tolerance. Remarkably, the application of low doses of impulses proved to be adequate in the treatment of the plantar nodules of two cases of LD. However, even in this case, a complete recovery of symptoms was not achieved. Again, another recent study [24] reported a treatment success rate of 70% (7/10 LD feet) at short-term follow-up and 80% (8/10 feet) at long-term follow-up by administering even lower ESWT energy densities, i.e., 0.10–0.14 mJ/mm^2^, 900 shocks, in 12 sessions at weekly intervals.

Compared to the aforementioned studies, in our study, we administered three sessions (once a week) of moderate-intensity-focused ESWT using 0.20 mJ/mm^2^. Although similar in terms of the administered energy flux density (EFD) to the protocol of Frizziero et al. [17], augmenting the number of pulses at 2000 shocks resulted in an increased total energy dose (TED) delivered during each treatment session [25].

EFD = amount of energy/surface area, measured in mJ/mm^2^;

Total energy dose (TED) = EFD × total number of impulses.

Furthermore, the frequency of shocks was set to 5 Hz, which led to immediate pain relief effects that appeared earlier than those that occurred in previous studies. Remarkably, this can be explained, at least in part, by blocking nerve propagation according to the gate-control theory. As a result, it is hypothesized that high-frequency shock waves may stimulate nociceptors to fire high-frequent nerve impulses more than low-frequency ones [27]. Again, several other molecular mechanisms by which ESWT alleviates pain in the musculoskeletal system have been proposed [28]. For instance, it is reported that the shock wave can distort the nociceptor cell membrane, avoiding the generation of membrane potential and, thus, the transmission of a pain sensation [29]. Another hypothesis is that ESWT can alter pain transmission by reducing the release of the pain-related neuropeptide substance P (SP) and other pain mediators from the treated area [28]. Furthermore, other authors speculated that ESWT-related pain relief could be attributed to the selective destruction of sensory unmyelinated fibers within the treated area, the activation of the serotonergic system, and/or the suppression of the pain system at the level of the spinal cord [28,30]. Despite these multiple possible influences on treating pain, further research is warranted to better define the precise molecular mechanism underlying ESWT-induced analgesia in plantar fibromatosis.

In addition to the aforementioned analgesic effect, our ESWT protocol produced a satisfactory response to the patient’s clinical symptoms and overall quality of life, as evidenced by the remarkable improvement in foot functional scores. Of note, our results are in line with previous case series reporting a softening of nodule consistency without any modifications in the nodule’s measurements [13,17,24]. Conversely, the US evaluation suggested that ESWT has been able to modify the appearance of the nodule. Despite morphological analyses not being carried out, we can posit that ESWT may be able to reduce nodule echogenicity by decreasing the collagenized fibrous bands. Evidence from both in vitro and in vivo studies demonstrates that ESWT’s application can improve healing by acting on the fibroblast [10,31,32,33,34,35]. For instance, Cui et al. [32] reported that ESWT improves the appearance and symptoms of post-burn hypertrophic scars by inhibiting the expression of TGF-β, which represents a master regulator of fibrosis, as well as that of other fibrotic markers such as alpha-smooth muscle actin (α-SMA), collagen-Ι, fibronectin, and twist-1. Again, ESWT was shown to improve scar appearance and pliability in patients with post-burn hypertrophic scars [33]. In another study, Haberal et al. [36] showed that ESWT application is immediately effective in preventing epidural fibrosis after laminectomy in rats. Therefore, considering these data, we hypothesized that ESWT administration may also exert a beneficial anti-fibrotic effect on plantar fibroma via the modulation of the TGF-β signaling pathway.

## 4. Conclusions, Limitations and Prospects

In recent decades, ESWT has received increasing attention for its potential beneficial effects on various bone and soft-tissue pathologies, yielding promising outcomes for pain relief and functional recovery. Compared to other conservative treatments or surgery, ESWT is a local, non-invasive, cost-effective therapy with no associated downtime, which allows the patients to return to their daily activities at any time following the treatment. In addition, it represents a safe and well-tolerated approach without severe side effects, complications, or surgical risks.

Increasing experimental evidence indicates that ESWT’s application can produce analgesic effects and facilitate fibrotic tissue remodeling, which may result in significant pain alleviation and improved functional outcomes in patients affected by fibroproliferative diseases, including Dupuytren’s and Peyronie’s disease. On the contrary, as far as we know, the literature regarding the use of ESWT for plantar fibromatosis is extremely scarce, and optimal application parameters have not yet been defined to ensure its effectiveness for this disease. This case report adds to previous limited studies in the literature and discusses the potential molecular mechanisms that underly the beneficial effects of ESWT for the treatment of plantar fibromatosis. Our case report confirms the efficacy of focused ESWT in reducing pain and disability and improving activity limitation in LD patients.

While acknowledging the limitation of reporting a case report, this study provides valuable data to guide clinical practice, showing that this therapy should be considered when choosing the best non-surgical approach for the treatment of plantar fibromatosis.

However, further research involving large-scale randomized trials and long-term follow-up is needed to determine the most effective EWST protocol for LD patients.

## Figures and Tables

**Table 1:** Roles and Maudsley score.

Grade	Rating	Interpretation
Excellent	1	No pain, full movement and activity
Good	2	Occasional discomfort, full movement and activity
Fair	3	Some discomfort after prolonged activity
Poor	4	Pain-limiting activities

**Table 2:** Changes in clinical and functional scores after ESWT.

Timing	VAS	RMS	17-IFFI			
			Pain	Disability	Activity Limitation	Global Score (%)
T0	6	3	23	36	0	34.7%
T1	2	2	12	7	0	11.2%
T2	0	1	0	0	0	0%

**Table 3:** Morphological evaluations of the symptomatic plantar nodule at different time intervals.

Timing	Diameter (cm)		Echogenicity
	Maximum	Minimum	Mean Grey Value
T0	1.46 ± 0.01	0.54 ± 0.01	0.87 ± 0.05
T1	1.48 ± 0.02	0.54 ± 0.01	0.84 ± 0.04
T2	1.46 ± 0.01	0.52 ± 0.01	0.50 ± 0.04 *

* *p* < 0.05.

**Table 4:** ESWT treatment protocols reported in previously published articles.

Refs.	Device	Application of Anesthesia	No. of Sessions	No. of Shocks	Frequency (Hz)	EFD (mJ/mm^2^)
[13]	Storz Duolith SD1	NLA	2	2000	3	1.24
[17]	Storz Modulith SLK	NR *	4	1600	3	0.20
[18]	NR	NR	3	2000	3	1.25
[24]	Evotron SwiTech	NR	12 ^#^	900	NR	0.10–0.14

* NR = not reported; ^#^ (mean 7.8 ± 2.9, range 1–12). NLA = non-local anesthesia.

## Data Availability

The datasets created and analyzed during this study are available from the corresponding author upon reasonable request.
